# E-prescription and invisible work in genomics in France

**DOI:** 10.3389/fsoc.2023.1152364

**Published:** 2023-06-29

**Authors:** Juliette Froger-Lefebvre, Quentin Lade, Estelle Vallier, Catherine Bourgain

**Affiliations:** ^1^Groupe d'Etude des Méthodes de l'Analyse Sociologique de la Sorbonne, Centre National de la Recherche Scientifique, Paris, France; ^2^Département SHS du Centre Léon Bérard, Lyon, France; ^3^Centre de Recherche en Cancérologie de Lyon, Université Lyon 1, Lyon, France; ^4^Institut Gustave Roussy, Villejuif, France; ^5^Université Paris Cité, Centre National de la Recherche Scientifique, INSERM, Ecole des Hautes Etudes en Sciences Sociale, Centre de recherche médecine, sciences, santé, santé mentale, société, Villejuif, France

**Keywords:** digitalization, genomics, electronic prescription (e-prescription), invisible work, cancer, high throughput sequencing, infrastructure studies

## Abstract

This article aims to analyze the transformations in medical prescription work and infrastructures brought by digitalization. Our fieldwork takes place in the context of precision medicine development based on genomics High Throughput Sequencing (HTS) in France, through the Plan France Médecine Génomique (PFMG 2025). The Plan aims at industrializing the production of genomic testing in clinical context at a national scale, particularly in oncology. To ensure the intensified flow of information between hospitals and HTS platforms required, a centralized process has been organized around two sequencing platforms and the introduction of a new e-prescription software (E-PRES). We start by analyzing how the e-prescription software changes the practices of health professionals by imposing new technological and professional standards. We show that, more than a mere prescription tool, this software is also a monitoring tool for the platforms and prescribers' work, and a support tool for the logistical and work organization. Secondly, we question the division of labor among the different professionals involved in the organizational or technical tasks required. We show that the feasibility of this new form of digitalized prescription relies on an important *data*work performed by “small hands” to select, translate and process a vast amount of heterogeneous data.

## 1. Introduction

The France Genomic Medicine 2025 Plan (PFMG2025) is a national policy organizing and financing the access to whole genome sequencing in care setting. All patients in the country, for which this analysis is deemed of potential clinical interest—either for diagnostic, prognostic or treatment- are eligible, notably rare disease and cancer patients. In this latter field, scholars have shown how the identification of specific mutations in genes such as BRCA, that strongly increase the risk of disease, has helped recompose medical nosology (Keating et al., 2016; Cambrosio et al., 2021) but also clinical work (Bourret, 2005). More recently, so-called somatic genetics turned toward the characterization of tumor cell DNA has undergone important developments. New treatments (targeted therapies, immunotherapies) whose prescription is conditioned on the presence of specific somatic mutations have been massively evaluated in clinical trials (Nelson et al., 2014; Polk et al., 2023) and commercialized. While some of these therapies are remarkably efficient, the current flow largely reflects an economy of promises (Hedgecoe and Martin, 2003) endorsed by both the drug industry and the regulation agencies (Salcher-Konrad et al., 2020). Inquiries on genomics in cancer care settings have highlighted the multiples forms that these practices of promises can take outside drug pipelines, in the patient' experiences and in professional work (Kerr et al., 2021). The reorganization and new division of work associated with the routinization of genomic tests has also been documented and analyzed as a consequence of the specific articulation work between clinical and molecular data, that this cancer drug related genetics entails (Beaudevin et al., 2019). Yet, the French Plan, with its focus on High Throughput Sequencing (HTS) technologies^1^, takes genetic analysis for the clinical care of cancer into a new dimension. In the field of rare diseases, Timmermans has proposed a rich analysis of the transformations in the genetic diagnosis work associated with HTS. He emphasizes the importance of new standards, in this work, namely international databases and the specific expertise required to use them properly (Timmermans, 2015). He also outlines the new ways in which these standards are articulated, through a collective endeavor, with both individualized molecular and clinical data to produce new forms of “causality for clinical purposes” (Timmermans, 2017).

Digital tools are a central part of this emerging HTS diagnostic process. In the case of the PFMG2025, if international databases are essential, a couple of additional tools have been developed among which a software, referred to by the actors as an e-prescription software, is of particular interest, given its role in the national access to HTS in different care context, including cancer. Digital tools have been used for decades in various fields of medical activity, including the organization of work with the so-called shared medical file (Lehoux et al., 1998), the financing of care with the National Health Insurance Interregime Information System (SNIIRAM) or the performance of medical acts with telemedicine (Mathieu-Fritz and Gaglio, 2018) or the algorithmic systems used to assist medical decisions (Anichini and Geoffroy, 2021). Informational infrastructure studies (Bowker, 2008; Bowker et al., 2010) have proposed insightful ways to consider computers, document scanning processes, software… Rather than “substrate systems” (Star, 1999; p 380), i.e. invisible backgrounds of work, these digital tool should be analyzed as infrastructures contributing to the organization of human work, with growing importance in situation where a variety of professionals performing tasks distributed over time and space are involved (Strauss, 1985; 1988). Some of this literature has renewed the analysis of “the ties between records and the social system that services and is serviced by these records” (Bittner and Garfinkel, 1967; Garfinkel, 1967; p. 192) and has highlighted the invisible work done by technicians to make the infrastructures operational (Shapin, 1989). This includes and shapes diagnostic work associated with intensive data entry, data care and logistical organization to ensure physical links between the stages of production organized from afar, that remains largely imperceptible to many members of the infrastructures when the device is working. Yet, this work of “little hands” requires specific skills (Denis and Pontille, 2012). While the updating of a database is a matter of expertise specific to so-called scriptural activities, its cognitive dimension has often been underestimated (Pontille, 2010). The invisible work behind databases consists of a set of tasks such as updating reports, cleaning fields, classifying, building a query, but also making the data compatible with the chosen digital format. These tasks must constantly be legitimized by the actors to show the extent of their work (Dagiral and Peerbaye, 2012).

The present article analyzes the implementation and the ways in which the new e-prescription software developed in the PFMG2025 is used. With this case study, we aim at contributing to the existing literature on HTS diagnostic work in care setting by introducing an analysis of the specific effects of digital tools on the organization of work and the forms of expertise involved. To do so, we draw on the informational infrastructure studies and take up their specific interest in the characterization of all forms of work, including the “invisible.” Invisible work is indeed concept aligned with descriptions of the underestimated role played by paramedical staff in diagnosis and categorization labor (Seim, 2022). While physicians are historically considered as the “traditional adjudicators of whether or not someone is sick” (Dumit, 2006; p. 576), other medical laborers, such as nurses or ambulance crews, also perform essential preliminary classification work.

## 2. Background

Inspired by similar foreign policies, such as Genomics England in the United Kingdom or the Precision Medicine Initiative in the United States, the French Plan differs from these research-oriented initiatives in its clinical ambitions. Initiated in 2016 and endorsed by the Prime Minister, the PFMG2025 promises a revolution in healthcare through a generalized access to genomic medicine (Bourgain, 2019). Practically, the Plan has financed the setting up of two national HTS platforms where the production of whole genome sequencing is centralized. All the patients in the country with a pathology identified by professional and regulatory bodies as eligible and with a clinician prescription, should get a HTS, performed on one of these platforms and included in their healthcare national coverage.

Started in the late 90′s with the first BRCA test (Bourret, 2005), the introduction of genomic medicine in the French cancer care has undergone a first acceleration in the late 2000′s when the National Cancer Institute launched a State-funded program to settle “platforms for molecular genetics of cancer” all over the country (Nowak et al., 2012). This initiative was an answer to the first market authorizations of drugs whose prescription was conditioned on the identification of specific mutations in the cancer cells. These platforms produced the required tests for all eligible patients in the country (Beaudevin et al., 2019). In parallel, implications in precision medicine clinical trials developed in a handful of expert centers, where clinicians accumulated a rare and recognized expertise (Besle and Schultz, 2020). Many of these clinicians have been involved in the design of the FMG2025 Plan and in its implementation in hospitals. Their central position in expert centers and the competition between specialized institutions helped getting support from most health professionals in this very structured clinical field of French oncology (Castel, 2008). Facing little resistance, the HTS prescriptions provided by the Plan are gradually becoming routinized.

In a previous work (Bourgain and Lade, 2022), we have studied the impacts of the new centralized organization set by the French Plan on the production of genomically informed medical diagnoses. We have described the work done by the actors to ensure the quality of these diagnoses, the involvement of historical actors and the place made for new professional entrants, notably the bioinformaticians. We also highlighted the decisive role the e-prescription software, showing that its usages went well beyond this sole act. The software, we claimed, actively contributed to the coordination of the complex sequencing pathways that goes from the initial prescriptions by oncologists to their final impacts on the patients.

The sequencing pathways generate a very large amount of data for a significant number of patients, that conveys an image of abundance medicine. The handling of the case flow from a diversity of hospitals all over the country required the setting of an *ad hoc* digital infrastructure. Following the guidelines of the PFMG2025, each of the two national HTS platforms (referred to as platforms A and B) developed their own system, and referred to them as e-prescription software. Although the two have specificities, they share important common characteristics. In what follows, they will be designated in a non-specific way, as E-PRES. In the present article, we analyze the specific issues raised by the production and use of digital data associated with E-PRES, with a focus on the initial prescription stage. While this informational infrastructure links the different professions and spaces involved in the genomic analysis production chain, as a technological mediator, it also translates and distorts information (Latour, 2007; p. 58). Its impact on the ways in which professionals organize themselves is therefore significant.

Our survey has been carried out at the peculiar and transitional moment of the implementation of these new genomic analysis pathways and associated computer system, as part of the PFMG 2025. We show that this centralizing intention clashes with the information systems and genomic medicine analyses that have been in place for several years in some expert centers. E-PRES is in competition with existing software in hospitals, and around which logistical processes and work organization of health professionals are well-established. In this context, clinicians have difficulties in appropriating this additional software and its interface, and they finally organize themselves to delegate the e-prescription related work. To this aim, they negotiate with reformers (as we propose to call the health professionals to whom the State has entrusted the implementation of the PFMG2025 Plan) the creation of new positions, mostly held by women qualified as “prescription assistants” or ensure that other professionals already present, such as genetic counselors, free up time to carry out the work of monitoring, updating and validating the various stages of treatment in E-PRES. At the time of our survey, the new positions were referred to as “prescription assistants.” Late 2022, their title was changed for “genomic pathway managers.” Using this type of profession to perform the dirty work (Hughes, 1997), corresponding to the administrative and time-consuming tasks performed by the bottom of the hospital hierarchy, is a common practice in the medical profession. Moreover, this invisible organizational work is mostly performed by overqualified women in often precarious jobs (Avril and Vacca, 2020).

Similarly, the work of prescription assistants seems to be invisible as it is carried out in the name of doctors and in a software environment that leaves little trace of their input, except when it is not performed correctly and the production chain is blocked. The moment chosen for our survey, during the phase of introduction of the E-PRES software, enables us to reveal the importance of this work done by “little hands.” Difficulties of appropriation by the clinicians, negotiations between professionals, training issues and the need to recruit professionals assigned to these technical tasks of prescription assistance are all indicative of their importance. In this context, our article examines the ways in which the introduction of the E-PRES software reaffirms the existence of little hands within the hospital, and more broadly within the medical order, and renews the forms of invisibilization of their work.

Box 1 **E-PRES: the different steps of the e-prescription software.**

E-PRES is a tool that collects patients' clinical data in digital and structured form: clinical signs, diagnostic informatoin, family history, digitalized consent form for genetic analysis. From this software, it is possible to initiate but also to follow the complex logistical process that ensures the transfer of a patient's biological sample from the local hospital where it was collected to the centralized HTS platform that performs the DNA sequencing and proposes a clinical interpretation. In the case of tumor samples, this process includes an additional step at an expert biology laboratory, where the quality of DNA is evaluated. Finally, once the HTS analysis has been performed and interpreted on the platform, the result can be deposited there, along with the associated clinical management recommendations. Many actors are involved in the steps followed by E-PRES, whether they produce the data it aggregates, use them and/or are concerned by the decisions it allows to be made.

## 3. Methods

The data used in this study were obtained from a field survey interested in the deployment of genomic medicine in France^2^, conducted from January 2021 to June 2022, in the two sequencing platforms of the PFMG (platforms A and B) and four hospitals or cancer centers in the Paris and Lyon areas. Given the COVID 19 pandemic context the fieldwork was conducted by different members of the team in the Lyon and in the Paris area. Previous fieldworks (on INCa platforms and on early phase clinical trials in several French hospitals) and contacts helped getting access to the platforms, hospitals and interviewees (see Table 1).

**Table 1:** Table of empirical material.

**Kind of material**	**Number**
Interviews with policy makers	3
Interviews on platform A	7
Interviews on platform B	5
Interviews with health professionnals	11
MTB observations	60

In the present article, we rely on qualitative data, i.e., semi-structured interviews based on grids adapted to the different interviewees and observations. Twenty six interviews were conducted with PFMG managers; staff recruited on the two platforms: managers, biologists, quality manager, medical manager; referring physicians and other professionals involved in the genomic test prescription chain, such as prescription assistants or genetic counselors. We started the fieldwork with interviews of three policy makers of the Plan, to get a general overview of the policy issues. Then, we first investigated the Parisian platform by meeting with the medical and operational managers who allowed us to interview the staff such as biologists or bioinformaticians. Although this entry through the management may have produced some bias in the information collected, it was the only way to proceed. In the sensitive moment of implementation, control over the information release on the platform was deemed as essential by the management. We proceeded the same way at the Lyon platform, first meeting the managers and then the staff. We were also able to visit the entire platform. Finally, the last part of the fieldwork consisted in observing sixty Molecular Tumor Boards (MTB) meetings in oncology, during which the selections of patients from three Parisian hospitals eligible for HTS were discussed. These observations were crucial for to identify difficulties linked to the handling of the E-PRES software and the decisive but often silent role of prescription assistants. Physicians, prescription assistants and genetic counselors were interviewed and a 1-day observation of the work of a prescription assistant was carried out.

Transcriptions of the interviews, subcontracted to an external provider, were analyzed with a double thematic coding and completed with the field notes from the meeting and workday observations.

Interviewees were given an information leaflet presenting the objectives of the survey and the conditions for data storage and processing. Finally, compliance with the CNIL's MR-004 reference methodology of the project was validated by the CNRS's Data Protection Department (DPD) (declaration n°220740). It also received the favorable opinion of the Groupe de Réflexion Éthique du Center Léon Bérard (GRET-CLB 2021-003) as well as that of the Inserm Ethics Evaluation Committee (CEEI-IRB00003888).

In what follows, we start by presenting the complex path of genomic analysis in a care context and analyze the ways in which E-PRES challenges existing practices. Secondly, we show that the implementation of E-PRES requires important adjustments on the part of clinicians and implies the work of “little hands” of new professions, to select and translate relevant data, sort documents and digitize the information required in the process.

## 4. The introduction of genomic prescription software in the French healthcare system

The introduction of e-prescription in care pathways linked to genomics appears to be a tool for controlling and transforming the nature of work. In this first part of the paper, we describe the multiple roles of E-PRES in the coordination, monitoring and organization of all stages of the prescription. The introduction of this new software, which coexists with already existing softwares and practices generates technical difficulties, but also frictions and negotiations between professionals. Finally, we discuss the transforming power of E-PRES, cognitive this time, on the prescription work itself.

### 4.1. Beyond e-prescription: a software for organizing and monitoring genomic testing

In order to understand the transformations caused by the introduction of E-PRES, it is necessary to look back at the complex pathway set up by the PFMG to carry out genomic analyses. Unlike the genomic tests carried out locally in some hospitals, platforms A and B centralize the sequencing of samples from the entire national territory. These platforms are thus located at a geographical distance from the professionals who order the analyses, and the various stages of the HTS test production are geographically fragmented. In this context, the care chain is distributed over more numerous and dispersed sites and IT infrastructures, primarily E-PRES, play a key role in linking and coordinating the different steps of the genomic testing pathways.

Within the framework of the PFMG 2025, the production of a genomic test is organized in several stages. The pathway begins with an initial prescription request from the clinician in charge of the patient's follow-up. This request is then sent to one of the 20 Molecular Tumor Board (MTB)^3^ labeled by the Plan throughout the country^4^, which assesses its eligibility before validating it. Eligibility has two dimensions. First, general clinical indications have been set by the Plan—in oncology, two broad categories: rare cancers and refractory metastatic cancers- and only patients corresponding to these indications can get an HTS analysis.

Second, the MTB also selects patients according to health indicators (number of metastases, “RMH” score^5^, etc.) and sometimes, more informally, according to other individual characteristics: age, social situation, location, but also according to the lifestyle and behavior of certain patients, such as whether or not they smoke. The collective decision made during the MTB meeting are based on the information collected in the patient's clinical file and made available from a software that is different from E-PRES.

Indeed, each hospital uses a specific software to handle the consultations or medical acts, the clinical information, evolution of the disease and its management, and the results of the biological or genomic tests already performed. Once the members of the MTB have collectively validated the HTS prescription, a new entry must be created in E-PRES that includes the prescription, clinical data and the patient's consent. This registration triggers the logistical and technical process of the genomic test. New software, specific to the PFMG pathways, E-PRES thus requires supplementary data work.

“*There, the patient is entered into [E-PRES] which is a software that allows the follow-up of different patients, and the interface between the different centers. From there, as soon as the patient exists in [E-PRES], we can ask the pathologist to transfer the tissues to the platform where the nucleic acids will be extracted. And so, there is again a bit of... I would say formalities, trying to mediate and coordinate between different people, with the pathologists, saying that this patient has been included. You have to send the tissue. The pathologists order a transporter. The tumor comes out of the tumor library.”*

*Interview with a neuro-oncologist, prescription physician, June 17, 2021*.

The samples are then sent to the sequencing platform, where various preparation steps are executed by specialized technicians. These final steps in the preparation of biological samples and sequencing require meticulous logistical development. The DNA must be extracted and prepared so that the molecules can be analyzed by the machines, the very high-speed sequencers, which are the cornerstones of the platforms. The sequencing of the samples is a particularly important step in the technical process: the biological material is transformed into digitalized information.

While other softwares are used during the sequencing process, E-PRES plays an important role at the end of these technical steps because it aggregates the genomic data analyzed by the biologists. Indeed, the subsequent steps of bioinformatics data processing require technical expertise developed by dedicated teams. The quality of the digital data generated by the sequencers is controlled before they are analyzed by biologists, using *ad-hoc* software, different from E-PRES, and developed by the bioinformaticians. Thus, E-PRES organizes the work of linking and articulating (Star, 1986; Strauss, 1988) data of different natures: genomic data, clinical data, legal documents such as the consent signed by the patient, but also external international databases containing, in particular, information on the role of variants. E-PRES is thus at the center of a complex, distributed information infrastructure of storage and computing, in which material and logistical issues play a key role. This makes it a priority for the manager of bioinformatics on platform B to have a system engineer in his team, who is able to operate and control the hardware infrastructure:

“*In fact, yes, my profile [...] that I like to recruit first, [...] is the system engineer. The one who will manage all the hardware infrastructure, the calculation server. Because without it, we don't really do anything. Without a good infrastructure that holds up and with good systems engineers who monitor and manage all that, you don't get very far. It's really the essential engine.”*

*Interview with Platform B bioinformatics manager, April 29, 2021*.

Beyond this function of organizing digital prescription, E-PRES is, for the platforms and prescribers, a tool used for logistical follow-up and work organization. Each step on the pathway is recorded in the software. The web interface displaying the list of patient files submitted for genomic testing indicates by a band at the top of the screen the number of files according to their status along the genomic testing chain: “waiting to be received,” “waiting for sequencing,” “waiting for results,” “completed.”

**Figure 1:**
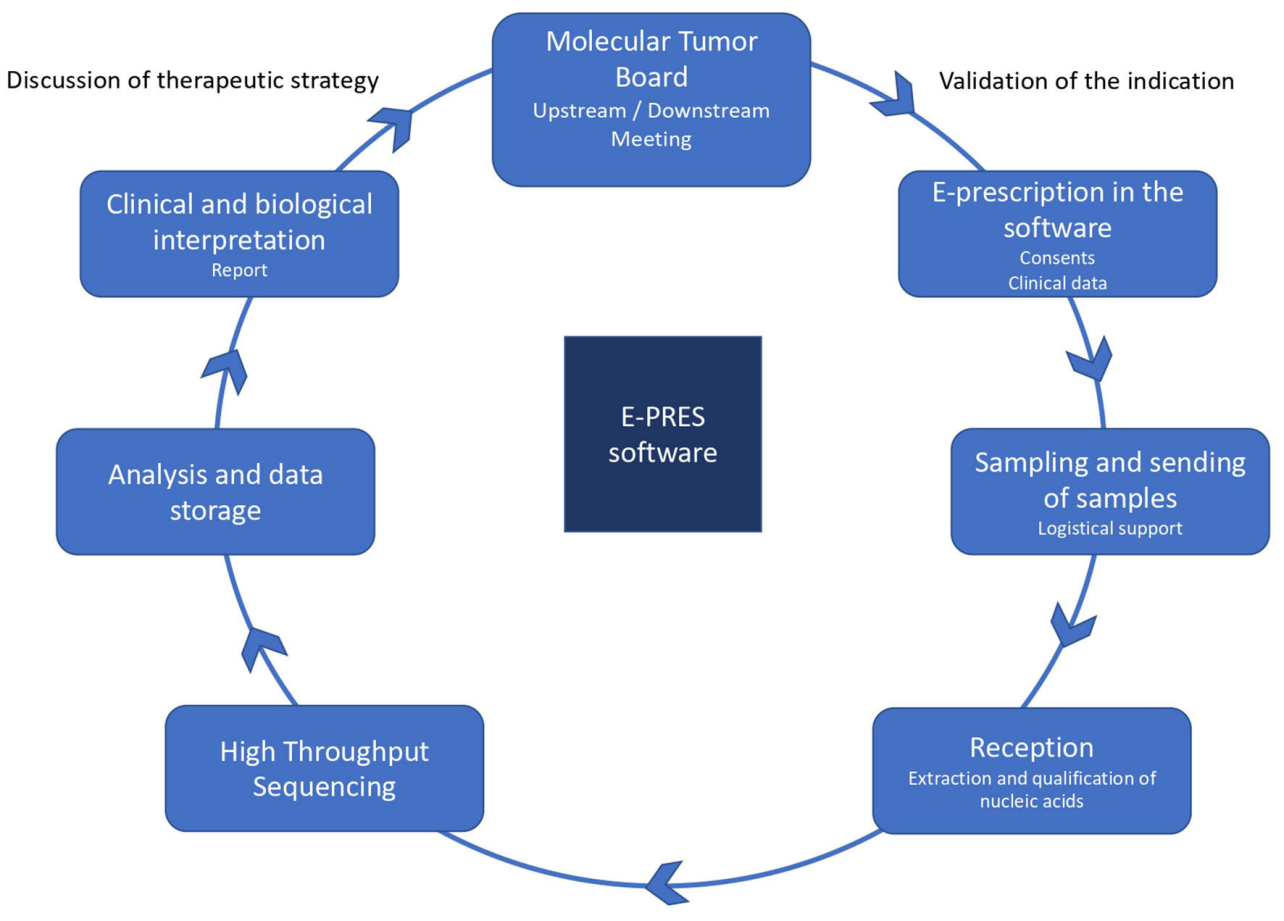
The prescription process for a genomic test. Source: https://www.oncorif.fr/professionnels/la-medecine-pan-genomique/.

Similarly, on the slides used to present the genomic pathway to professionals, E-PRES is located at the center, in position to follow all steps, from the patient's registration at the MTB to the report of the clinico-biological interpretation informed by the HTS results (see Figure 1).

“*So, we have [E-PRES], which is the electronic prescription system that actually becomes our control tower as well. We manage many things within [E-PRES]. That is, it's not just the prescription. It goes all the way to the report. The report is deposited in [E-PRES]. So, it allows us to track by theme: the physician has prescribed, the tubes arrived in the laboratory, the sequencing is done, the computer analysis is in progress… That's it. It allows us to follow the evolution of the sample, basically.”*

*Interview with Platform A medical director, April 06, 2021*.

E-PRES is thus a tool designed to meet the organizational, technical and scientific constraints generated by this centralized HTS production scheme, through its ability to link spaces, hospitals and platforms, and professionals who are geographically distant, but also more diverse and more numerous.

### 4.2. The centralizing effect of E-PRES added to the localized systems already in place

In addition to its function as a tool for logistical follow-up and organization of the prescription work, E-PRES also has a role of centralizing information. Yet, as E-PRES is superimposed on existing local solutions, specific work is required to adapt to its interface and computer input. The changes in practice required are a source of resistance from certain clinicians, negotiations between professionals and, consequently, frictions (Beaudevin et al., 2019).

The PFMG 2025 aims to generalize and harmonize access to genomic medicine. In this context, the E-PRES software allows the platforms to be electronically linked to referring physicians located throughout France. As the management of platform B explains, the particularity of their sequencing laboratory is that it is entirely dematerialized and goes beyond the boundaries of the hospital:

*Platform B medical manager: “You can't go to something like that without having a tool for prescription. So, we had to... the lab is necessarily totally dematerialized. So only that, a basic lab, it gets tubes with erasers and pencils. And we can manage a lab without having a computer system. Here, we had to totally... yes, dematerialize the entire process.”*

*Platform B Operations Manager: “Compared to a regular hospital, also there was the fact of going beyond departmental boundaries. In other words, whether you're in Avicenne, Bichat or Necker [three hospitals in Paris], you can send and get a result for your patient. So... and whether you are in Rennes or Lille too. So it places everyone on an equal basis [...] That is to say, if you are in Rennes and you have three patients, you will send one here, one there... So there, it is still possible to centralize.”*

*Interview with Platform B management, May 11, 2021*.

E-PRES has been designed to be directly used by prescribing physicians before MTB using personalized identifiers and codes. This information can be completed later, during and after MTB's discussions. However, handling the software involves additional work for clinicians: they have to get used to its interface and functioning. An important characteristic of E-PRES is that the progression throughout the different prescribing steps is conditioned on the presence of specific digital information, namely patient consent, clinical information and genomic analysis reports. The digital data management work is thus crucial to allow the progression of the prescription process.

“*E-prescription is something new. It's a bit of a novelty in this Plan (...) Perhaps we will first make sure that they understand the tool. Because it's true that imposing e-prescription on a thousand physicians... We have very different generations. But it's true that e-prescription is a bit of an irruption in the world of some clinicians who were used to filling out notes. (...) So it's in this temporality. A bit of an immediate aspect. It's that: I have my patient and I prescribe.”*

*Interview with Platform B medical director, May 11, 2021*.

Moreover, E-PRES is an addition to and not a replacement for the pre-existing local software specific to each hospital. The medical manager of one of the platforms explains some of the blockages and resistance from clinicians by the “cumbersomeness” of the operations and the work involved in appropriating the software:

“*For cancer I think, one part that is blocking is the cumbersomeness. That is to say, we had to create an electronic prescription software called [E-PRES]. Well, like any new software, you have to get used to it, and it takes some time to make an electronic prescription.”*

*Interview with Platform A medical director, April 06, 2021*.

This implementation “cumbersomeness” also stems from the centralizing role of E-PRES, marked by technical difficulties in making the different software programs interoperable. Indeed, E-PRES has the function of monitoring the entire genomic test process and the correlated gathering of clinical and biological information. However, on the sequencing platform, part of this information is first managed through two other software, one used to monitor the biological sample preparation and the other to help interpreting the sequencing data. On one of the platforms in particular, these two software are not interoperable with E-PRES. Similar technical difficulties exist with local hospital prescription software. In this case, making the different local prescription software interoperable with E-PRES is not considered as an option by the actors.

This brings us back to the problems already studied in relation to the implementation of systems which must be made compatible with systems already in place, and for which the work of unifying and upgrading the information takes a great deal of time (Bowker and Star, 1999; p. 107–108). Indeed, the clinical information of patients, already present in the local prescription software specific to each hospital, must be adapted and re-uploaded in E-PRES by the professionals. This requires, in addition to the appropriation of a new tool, additional time for entering patient information, which is perceived as too great a burden for some of the prescription doctors. For example, a clinician involved in setting up one of the platforms described the discussions she had with the bioinformatics manager responsible for designing the e-prescription software.

“*In fact, at the beginning, [the bioinformatics manager] wanted the doctor on [E-PRES] to rewrite the entire form. I told him: in fact, we'll have to do the form on [the hospital's software], then we'll have to redo the form on [E-PRES]. I said [to the Bioinformatics Manager] it's not possible, in fact. So, you have to delegate the access rights to a secretary or a genetic counselor. Because if you don't, the doctors aren't going to do it. So, we don't have the time, I tell you I don't have the time. So that's it. So that's not possible.”*

*Interview with a physician oncologist in charge of a Molecular Tumor Board of the Plan, January 12, 2022*.

This quote is revealing of the type of negotiations between professionals on the modalities of appropriation of the technology and the accomplishment of the prescription work in digital form. Although this was not the case at the beginning of the implementation of the genomic test process, prescription assistants and genetic counselors have been hired to support the clinicians in the digitization of prescriptions. Yet, prescription in E-PRES requires a specific work to translate clinical data into the standardized categories implemented in the software, which generally implies the clinician's expertise.

### 4.3. Translating clinical data into standardized and computerized language

The digitization of the clinical information collected transforms it by imposing a standardization of the clinical data. This requires cognitive work by professionals who must translate clinical observations into a standardized form. In local prescription software, while some of the patient information is entered directly into the interface, a significant portion of this data is made accessible to professionals through the input of scanned documents into the software. As Garfinkel (1967) has already shown about non-digitized medical records, although the complex documents included in it prevent a form of standardization of the data, they do not distort them, so that they can be used in future, yet-unpredicted, clinical contexts. On the contrary, E-PRES is designed to ensure that patients' clinical information is entered in a standardized form, according to international HPO (*Human Phenotype Ontology*)^6^ codes developed to categorize disease phenotypes. They can then be manipulated directly by data processing algorithms developed by the bioinformatics platform.

“*The prescription is really the entry point and we can collect a lot of information and data that are very important. [...]. On the genome, it's quite brilliant because as soon as I need data of interest that could be useful for sorting efficiently and automatically in [the other software], I just add boxes in [E-PRES] and I ask the clinicians to fill them in. And in that way, I know that I have this data that is well structured in the nomenclature that I want and I reuse it afterwards directly.”*

*Interview with the bioinformatics manager of platform B, April 29, 2021*.

The coding of patients' clinical information requires a long but also skilled work of analytical rereading of the available clinical elements. It is a matter of making the necessary decisions to decide on the most relevant codes.

“*There are still [...] 15,000 HPO codes. Yes, it's not 3, there are 15,000. So, it's true that today, either you don't put in enough and you're lost. Or, you put too much and you drown the fish. So, what we're going to try to find out is that there is a middle ground in describing exactly what the patient has. There is a prescription issue.”*

*Interview with Platform B medical director, May 11, 2021*.

These issues of arbitration on the information that should be coded in HPO format are a form of “tacit knowledge” (Collins, 1974) specific to clinicians. It is not just a question of transforming information into numbers; the expertise of the clinicians is essential in order to identify the clinical data that will be relevant for the type of analyses planned. Moreover, a specific and collective reflection on the quantity of clinical data that must be provided, is carried out jointly by the prescription clinicians and the biologists interpreting the genomic data.

“*Before, when we did a prescription in [E-PRES], it was frozen. It was blocked. When it was done, it was done. Whereas today, we have been able to block the identifying band [of the patient]. His consent, last name, first name, date of birth, etc. We can continue to enter clinical data as long as the data is not being analyzed. Therefore, we set up what we call CBIR, Clinical-Biological Interpretation Meetings. We have almost finished. So, it's during these CBIRs that we can collectively say: this file should be enriched on a clinical level.”*

*Interview with the medical manager of platform B, May 11, 2021*.

The HPO codes modify the prescription work because the coding implies a specific reflection of the health staff on the choice and the number of relevant HPO codes to select. E-PRES has a mediating role in this work by organizing the interactions between clinicians and biologists. This additional work thus highlights an important transforming effect of the informational infrastructures. From the point of view of the designers of one of the platforms, the objective is that the prescription work should be modified during consultations so that the physician writes it directly according to the HPO standards and, in the long run, it will no longer be necessary to translate the prescriptions according to these standards.

*Manager of platform B: “So, we arrive and suddenly we tell them: “Guys, we're sorry, we have standards now. Okay? You have to stop sending us the exam, whatever it is, and then take this and work it out” [...] You have to write a prescription at the consultation that is as efficient as possible in terms of... data. Because it's the clinical description of the patient that is going to be essential for the interpretation.”*

*Interview with Platform B medical director, May 11, 2021*.

From the point of view of the same designers, beyond the quality of the prescriptions for each patient, it is a question of creating a database in which the clinical information can be directly manipulated by the data processing algorithms developed by the bioinformatics platform to handle sequencing data. Yet, as noted by a designer of E-PRES, this touches upon a crucial expertise of clinicians that is central to their diagnostic work and might be difficult to share.

“*I don't know if I should say this, but [...] we have a whole area of clinical research that has been built on identifying the causes of genetic anomalies in their patients. So, it's true that we are dispossessing them a little bit... Well, if they have to put all the data in a shared database, there's a little bit of... I don't know if it's dispossession but... there's a sharing aspect that is actually quite easy in IT and quite easy in biology. It is perhaps less easy in the clinic. So, there it is, it passes time.”*

*Interview with Platform B medical director, May 11, 2021*.

It is thus the core of the prescription work that is transformed under the effect of the new importance of informational infrastructures, which are now central to logistics organization, but also to more directly cognitive work functions. In a seemingly contradictory way, this system of standardization of clinical data in HPO code, appearing to be a highly automated process, actually increases the evaluation and selection of clinical data as well as the work of adjustment between clinicians and biologists. This digitalized “precision medicine” systems increases the amount of laborious technical work involved and, as we see in the following, of feminized dirty work in particular.

## 5. Supporting digitalization: the invisible work of a genomic test prescription

Material, organizational and cognitive issues are at the heart of the prescription process and, as we have just seen, the digitalization of this process requires adaptations from the clinicians, centered on the integration of the E-PRES software into their practices. Yet, the appropriation of this software is not limited to the work of clinicians but is accompanied by an intensification of the dirty work (Hughes, 1997) carried out by the non-medical staff. Indeed, besides the training sessions implemented by the PFMG to facilitate the clinician's use of E-PRES, they are also supported by new professionals in charge of invisible tasks.

### 5.1. Training and informing on the use of e-prescription

In order to support the learning process of E-PRES, the two platforms have set up webinars. The multiplicity and geographical dispersion of clinicians likely to prescribe throughout the country have led to a digital version of these training sessions. Clinicians can therefore follow them at distance and have access to a prescriber's user manual. The objective of these webinars is 2-fold. If the modules aim first at acculturating clinicians to E-PRES, they also condition their access to the latter. For one of the platforms in particular, it is only after following the tutorial and answered two multiple-choice questionnaires that an account can be created for the clinician:

“*So, they [the physicians] do a little 30-min learning session. And they answer two quizzes. Once they have answered… So for the moment it's still manual because... [Laughs.] We receive the answers by email [...] And once we have the answers, well, if they have more than 60% correct answers, well… On the two MCQs, in general they don't have too many difficulties. So, we ask them... well, we create an account for them. And then they can start their first prescription on the tool.”*

*Interview with the operational manager of platform A, March 10, 2021*

However, these distant training are not enough to get prescribers to use the software. Indeed, the implementation of E-PRES requires a succession of very physical meetings between peers to explain what it is and how it works. We find here the presence of “active relays” (Benedetto-Meyer and Boboc, 2019; p. 101) already studied in the world of private enterprise that accompanies digitalization. In the implementation of the PFMG, several actors have made the choice to familiarize their peers with digital tools and the new prescription process in the broadest sense. This need was felt by the staff of one of the platforms who noticed that prescriptions were made on the software but that they did not receive any samples.

“*And we noticed that in some centers, there were prescriptions but afterwards the sample never arrived on the platform. So, we wondered about this. And what was happening ? The referring doctor was saying: well, I clicked, so it's good. Except that no one was informed that the sample had to be sent, etc. So, there was no way of knowing what to do. So, there was no internal follow-up. So that was one of the points that we regularly made. And in 2019, we asked all the medical oncology department heads to attend. We gave very theoretical presentations on what is the project ? How does it work? What should be done? And then, on request, [...] we did quite a few TC [teleconferences], presentations, but in restricted committees, with a CHU [public hospital] problematic, by center, in fact. So there, we did it more on request in a way to show them examples of what works and what doesn't, and how we could help them. And I think it was beneficial because we can see that for some university hospitals, there were many requests that had been made and that have been abandoned.”*

*Interview with the operational manager of Platform A, March 10, 2021*

In this case, the care pathway of the PFMG was not already embedded in the hospital organization and the physician was supposed to accomplish all the logistical steps by himself. This situation explains why nothing happened after he validated of the prescription by the MTB.

On the two platforms, the implementation of the prescription pathway requires presentations of the software and its associated steps so that physicians in each hospital can appropriate it. These training sessions are also provided in structures that could support physicians in addressing these issues, such as the Regional Health Agencies (ARS)—the institutions responsible for implementing the State's health policy in the regions -, existing cancer networks, etc.:

“*I do a little less [presentations] but here, for example, next week I have an appointment with the ARS [Agence Régionale de Santé] [...] next to Tours, where we have not succeeded in setting up the MTB. To try to stimulate this kind of things, to speed up the operation. When there is a problem in the regions, as soon as there are questions [...] I do coordination work. [...] Last year, in June, I even went to make a presentation of the Plan. It was at the [regional cancer network bringing together several hospitals]. I also made a presentation in the North of France. [...] Afterwards, my name is still around because at the beginning, I was the only one to train everyone on [E-PRES].”*

*Interview with an oncologist, head of a molecular tumor board (MTB's) for the PFMG, January 12, 2022*.

This assistance to digitalization through trainings or presentations is carried out either by clinicians involved in the development of the PFMG or by people with positions of responsibility in the platforms or in hospitals. Yet, these measures (webinars, training, presentation) are not sufficient to overcome the clinician reluctance to use E-PRES. Using the software is also time-consuming. A new division of labor has been set, that involves the recruitment of new professionals in charge of all extra tasks associated with the e-prescription.

### 5.2. The emergence of new prescription support professions based on the clinical research model

While the use of HTS technologies by the PFMG is new in the healthcare setting, several hospitals have already integrated in-house molecular screening programs based on restricted forms of genetic analyses, i.e., own gene panels, into their clinical routine. Thus, E-PRES is not only an addition to existing softwares, but it also fits into existing genomic medicine practices. In this context, E-PRES participates in the appropriation of new genomic technologies for care, which are supposed to allow a particularly fine analysis of the patient's genome. Moreover, the software centralizes the follow-up of the different steps of the genomic test and is thus supposed to simplify the monitoring of the results.

In some cases, however, the implementation of E-PRES has been hampered by these internal processes, which are considered more efficient than the prescription chain provided by the PFMG. Indeed, at the time of the survey, the HTS on the national platform was carried out in longer delays—3 months in the best of cases—than in some health care institutions, where the HTS pathway took about 2 weeks. For some physicians HTS precision level and its associated delay could thus be deemed inefficient for the patient care, as one of them explained in an interview:

“*Because if you look at it, the hospital administration tells you at the same time: send your samples to [platform B] and don't do your research here [within the hospital]. So, they don't understand that we do a targeted search and we have the result in 15 days. So, we can give the result right away. [Platform B] currently takes between 3 and 4 months. So, for a disease that has a median survival of one and a half years. [...] So there are a lot of problems at that level.”*

*Interview with a neuro-oncologist, prescription with E-Pres, June 17, 2021*

For the cancer with a life expectancy does not exceeding a few months, the sequencing times erquired by the PFMG platforms are not compatible with clinical needs. Some patients die before the results of the Plan's genomic tests:

“*For now, there is an observation phase to see how it works. And then after that, what happened to the delivery of the results. Now I think we have the results for four or five patients. Unfortunately, I know that the results were returned... because at least two of my patients had died when we got the results. So, I didn't have the opportunity to give them back the results [of their test].”*

*Interview with a professor of neurology, prescription with E-Pres, June 10, 2021*

The match between the temporality of the platforms and the temporality of care is crucial for genomic pathway to succeed (Beaudevin et al., 2019), and the question of delay is thus a major improvement issue to satisfy clinical needs. The shortening of the latter relies both on the recruitment of more bioinformaticians and biologists available for the analysis of an increasingly large quantity of data (Cambrosio et al., 2021) but also on an increased monitoring and optimization of the sequencing process.

In this context, most of the clinicians who are familiar with genomic medicine, and already involved in clinical research activities, intend to reproduce their work methods in this scientific framework. Indeed, the proper organization of clinical research trials relies on Clinical Research Associates (CRAs) whose job is to ensure the monitoring of protocols and the quality of data collected from the investigating physicians, i.e., those responsible for the trials (Petit, 2018). Similarly, the role of “little hands” appears essential here, not only to carry out tasks considered as time-consuming by clinicians but also to optimize processing times between each stage, validations, negotiations. Clinicians have gradually imposed the idea of using positions similar to CRAs to ensure the prescription of a genomic test in E-PRES:

“*And that's where we felt the need for the prescription assistance. Because they [oncologists] are not used to do that. They are used to work with the CRAs [Clinical research associates], with clinical research technicians. In fact, oncologists, when they include, [...] we prescribe. Because here we prescribe diagnosis and therapy. So, we are in the context of care. And it's true that it's complicated because we're in the context of care, so we don't need the resources for clinical research to support it. But the problem is that we face physicians [...] who don't know how to fill out this type of document because they don't usually do it. Because usually the CRA takes care of that. So that's why we've created positions called prescription assistants that can help physicians. But the validation remains the responsibility of the physician. Because it is under his responsibility, his prescription must be made. So, it's true that these prescription supports, I felt this need. But we didn't have the fundings, it wasn't foreseen in [the platform project]. In the case of the rare diseases, they found the solution: they have it financed by the Rare Diseases Plan. We did not have this, because there is no such plan for cancer. So that's why in the CLCCs [centers] or the university hospitals they now send some CRA staff to help the doctors.”*

*Interview with the operational manager of platform A, March 10, 2021*

However, the funding of these prescription support positions was not anticipated by the PFMG2025. Consequently, as the operational manager of one of the platforms explains, some CRAs had part of their position re-assigned to support prescription physicians on E-PRES. In some hospitals, genetic counselors provided this prescription support. Ultimately, thanks to a supplementary financial envelope released by the Plan FMG, dedicated prescription assistance positions have been created in several hospitals. This heterogeneity of professionals accomplishes an invisible but necessary work for the prescription process to run correctly.

### 5.3. The invisible “little hands” of genomic medicine

One of the unexpected effects of the digitalization of prescription is, in particular in the case of the genomic tests provided for in the PFMG, the need for greater technical supervision (Carricaburu, 1994), in the sense that prescription now includes various obligatory steps that did not exist until then.

*Interviewer: “Can you tell me exactly what are all the extra steps?*

*Doctor: Entering consent. Print the prescription for the blood draw. Call the transporter to have the blood*

*samples sent [to the platform]. Notify the anaphylactic physician that he must send the samples…[…]*

*Well, It's... it's heavy. It's very, very burdensome, in fact.”*

*Interview with a physician oncologist in charge of one of the MTB including patients in the PFMG, January*

*12, 2022*

This higher complexity of the prescription chain increases the technical supervision needed, particularly for the validation of the different steps in the monitoring software, for which the prescription physicians are originally responsible. Moreover, the work of entering the patient's clinical information into the software does not always end at this point. The clinicians may be recontacted if an error or inconsistency is detected during the following stages of the test, at the sequencing platform or by the biologists. During an interview, the manager of Platform A mentions a discrepancy in the sex of the patient between the data in EPRES and the biological sample analysis on the platform:

*Lab Manager: “In fact, it's not an F [female], it's an M [male]. [Laughs] Here again, there is a mismatch between what was entered by the referring physician and what we see behind it. And well, we have to inform the physician. Well, it's not up to us to modify the prescription data*.

*Interviewer: And for that, very concretely… You make a phone call?*

*Lab Manager: Yes, I mean, I make a phone call and send an e-mail, saying: “Well, we see that we have this, so would you...”*

*Interviewer: And you do that?*

*Lab Manager: No, not me directly. The biologist or the intern who actually does all this correspondence, saying: “Well, we saw that. So, can you modify...?” Because as a result, it's under the prescription that you have to modify the gender to regenerate the right data. And then, the bio-info will say: “ah well, it's a male and we can see a male, so everything is fine.”*

*Interview with Platform A Lab Manager, March 30, 2021*

This excerpt reveals the decisive role of the “invisible technician” in laboratory work (Shapin, 1989) and, more recently, the cognitive work required to update medical databases (Pontille, 2010). However, faced with the diversification of administrative tasks assigned to clinicians, the digitalization of prescription imposes, alongside laboratory technicians, the arrival of new professionals who support clinicians, thus participating in the process of stratification of the health professionals (Freidson, 1988). Essentially composed of women, these new professions, often considered to require less technical training, are at the bottom of the chain of delegation of medical tasks, representing a division of medical work that gives pride of place to the most qualified tasks, and therefore the most prestigious (Arborio, 2012). Indeed, the implementation of e-prescribing software reinforces gender biases that the literature has already well identified in several countries. As such, Elianne Riska and Katarina Wegar's sociological survey of health care systems in India, Great Britain, the United States and Finland shows the segregation in the division of labor where the work done by women is devalued, while the work done by men is highly valued because the professions define it as work with measurable skills (Riska and Wegar, 1993).

On the one hand, the prescription assistants, who will be employed by the reference centers for the prescription of the genomic test provided in the PFMG, are more often called upon for administrative tasks directly linked to the process of digitalizing the prescription. On the other hand, other professions, also dominated by women, support doctors during certain stages of the prescription process. This is the case of genetic counselors who intervene, in our case, at the stage of signing consent by patients. On the model of the clinical research assistants solicited in the framework of therapeutic trials as logistical support, the genetic counselors find themselves between care and research, having to master certain technical knowledge in order to explain it to the patients and to support the doctors in their administrative tasks, as one of them explains:

“*I work with three doctors. So, I'm under their delegation, legally speaking. So, I prepare everyone's consultations. Mine and the doctor's. So that means getting the medical records. Sometimes asking for tests beforehand so that we can move forward. Asking the patient for information on his family... also preparing his family tree in advance. Then there is the whole consultation part which is the biggest part of the work where we receive the patients in consultation. We establish their family tree. We list all the personal history. [...] So once we have explained all this, if the patient wishes to launch this genetic testing process, we sign a consent form. So, we explain to him the interest of the test. And what he will be legally obliged to do, including the obligation to the family. If he agrees with all that, he signs the consent.”*

*Interview with a genetic counselor, January 7, 2022*

Despite the centralization and digitalization of the monitoring of the prescription chain via e-prescription software, this supervision is largely based on the work of “little hands” (Denis and Pontille, 2012), which are invisible and not very highly valued by the health professionals institutionally responsible for validating prescriptions. This is a form of “dirty work,” not very prestigious because it is highly administrative, and because it is delegated to new professions located at the bottom of the delegation hierarchy (Hughes, 1997). The links between dirty work and gender have tended to focus in the literature on nurses and care assistants, who perform tasks that are considered less rewarding than those performed by doctors (Bolton, 2005). In our case, women occupy positions as administrative assistants in order to run the software. Nevertheless, both prescription assistants and genetic counselors are overqualified (“master's degree”) for the “dirty work” they perform. However, they do not have the skills to perform all the work assigned to the physician. They do not have the medical expertise required for the diagnostic work, they are consequently relegated to the less qualified tasks of prescription work, such as administrative tasks. In fact, this work requires little technical training, no interaction with patients and, finally, little recognition by the institution itself. Some of their tasks on the software require them to use the clinicians' identifiers and passwords. Despite their daily involvement in the digital tool, they have only a restricted official access to it, despite its importance to the proper execution of the job. This contributes to the lack of consideration for their status and the invisibility of the indispensable nature of their work. Indeed, since access to some of the software's functionalities is restricted, they often have to borrow the login of a doctor or a manager to be able to perform follow-up or reporting tasks. As a result, their name does not appear in the tracking of activities performed. Using someone else's login and password strongly echoes the tasks that lead individuals to the rank of “non-persons” (Star and Strauss, 1999), especially since they do not appear in the reports they have written.

This invisibilization can also be explained by a restricted conception of prescription time made necessary by the digitalization of its processing. Clinicians have only partial visibility of the stages of e-prescription, whereas behind the scenes, the extended time of prescription must be considered. The digital version of prescription requires a multiplicity of administrative procedures, both upstream and downstream of the molecular tumor board. Indeed, prescription assistants must ensure that all the information necessary for the discussion of the patient's case appears clearly in the software form. During a day of observation of the work of a prescription assistant, we were able to record in our notebook all of these information gathering tasks:

*In the case of patient 1, the prescription assistant shows us that an information is missing in order to calculate the patient's RMH score, which is essential to determine whether or not the patient is eligible for the genomic test. The file remains on stand-by until she can fill this gap, by contacting the referring physician to ask for more informations or his medical secretary to get new documents from the patient file*.

*Excerpt from the observation notebook of a day's work by a prescription assistant in a health care institution in Paris, conducted on 22 March 2022*.

The work of prescription assistants can also consist in providing guidance to the physicians and checking that all the information is present. This means entering all of the new prescriptions into the software, requesting the removal of biopsy samples which may be in remote institutions, organizing the safe transport of blood samples and finally launching the analysis. It is also a matter of notifying the DNA extraction centers and the anatomopathologists of the new samples in progress. Finally, the bulk of the work is done around the tracking and the execution of tasks that are supposed to be done by the prescribers (physicians): creating a patient number in the software, retrieving the signed consents, filing the necessary documents, and validating the prescription in the software. A prescription assistant told us, “*Physicians don't go to [E-PRES] to look up information: where the sample is and so on. It is not at all practical for them*”^7^. It is therefore a question of doing “what remains to be done” (Avril and Vacca, 2020; p. 89) and what the doctors have not done themselves, covering both the failings of other professional groups and those of the software itself, which is supposed to ensure the monitoring and the centralization of data.

Prescription assistants are in charge of monitoring and, above all, of registering the e-prescription. They carry out a whole range of invisible tasks, which go beyond the physical framework of their office and which only become visible when they are not carried out, such as reminders of appointments (Avril and Vacca, 2020) with the doctors who prescribe for the signing of consent. Their work is made invisible by the very tool of the e-prescription software, which is supposed to carry out all these tasks by itself, which ultimately becomes the responsibility of the prescription assistants. On the other hand, if this work is not carried out, it is made visible insofar as the implementation of the software has revealed the need to recruit people to make it operational.

## 6. Discussion

The Plan FGM2025 embodies the centralized vision of the French State in supporting the implementation of HTS technologies in healthcare. To be effective at the entire national scale, this vision a radical increase in the standardization and digitalization of prescriptions. Consequently, E-PRES appears as a central tool in the PFMG2025 large-scale implementation of genomic in healthcare. The software has a decisive organizational role in the design of new care pathways and in the work division between professionals to integrate the HTS genomic technology into their practices.

In addition to collecting and digitalizing information, E-PRES help monitoring the care process, linking and guiding each stage of the genomic test. Digitalizing the prescription by means of software should allow any physician to refer a patient and guarantee the principles of equity for patients.

Nevertheless, this desire of health system regulators to impose a new organization of care at a national scale is confronted with logistical and technical difficulties as well as with pre-existing local practices within hospitals. The time required to perform the genomic test proposed by the PFMG is longer than those routinely performed in hospitals. Further, while the software is taking on a central organizational importance in the care pathway, it also requires much more specific work.

The PFMG's attempt to involve clinicians in this procedure of long-distance prescription encountered resistances directly linked to the hospital division of labor. In the end, the implementation of genomic medicine pathways turned to be in line with the pre-existing practices of the medical profession, namely the use of “small hands.” Thus, far from dematerializing care, E-PRES has largely intensified the “dirty work,” most often assigned to women, who are poorly recognized, in precarious positions (fixed-term contracts, part-time work, etc.) and often overqualified. This work is also largely invisible because it is integrated, without leaving any trace, into the routine usage of the software. Yet, this work is crucial for the proper running of these pathways and particularly for the respect of deadlines. Ensuring that the software procedure are completed is essential if the HTS genomic analyses should improve care for patients who are often in precarious therapeutic situations.

## 7. Conclusion

This article analyzes how the digitalization of prescribtion and the organization of health professionals associated with precision medicine are transforming medical work. Far from avoiding any human intervention in the process, e-prescription requires the use of digital tools whose manipulation relies on learning and adaptation, but also on inputting, translating and sorting information. The automation and ddematerialization brought by software such as E-PRES, also increases the burden of work done by small hands, very largely feminized.

Largely made invisible, these tasks could qualify as dirty work. Yet, given the specificities of the clinical context and the operations of translation from paper to digital (or from one software to another) that are required, they should imply a certain form of expertise. We have described the centrality of small hands in the functioning of E-PRES but many of them do not have a formal nominal authorization to access it. Further, the software does not keep track of the data entry and formatting work and. Consequently, our enquiry did not allow us to observe the expert work of the little hands in the making. To further describe the effects of digitalization on the work of prescription, additional fieldwork with longer observations of the little hands daily work will be required, with more systematic observations of each task carried out, from data entry to bioinformatics, sorting work and monitoring of the prescription process. This would allow a more specific analysis of this prescription work nature, from the diagnostic work to the announcement of the results to the patient. We would be able to question the relational dimension of diagnostic work (Seim, 2022) not only between the different health professionals, but also between human work and that of the algorithms for labeling clinical data, in the particular case of the little hands and the prescription software whose operation they are responsible for.

## Data availability statement

The original contributions presented in the study are included in the article/supplementary material, further inquiries can be directed to the corresponding authors.

## Ethics statement

The studies involving human participants were reviewed and approved by CEEI/IRB Comité d'Evaluation Ethique de l'Inserm: The ethics évaluation committee of Inserm, the Institutional Review Board (IRB00003888, IORG0003254, FWA00005831) of the French Institute of medical research and Health, has reviewed and approved the research project. The patients/participants provided their written informed consent to participate in this study.

## Author contributions

All authors listed have made a substantial, direct, and intellectual contribution to the work and approved it for publication.

## Footnotes

^1^This is high-throughput sequencing that allows the analysis of the entire human genome, and no longer only a panel of genes targeted and previously identified by biologists.

^2^This survey is carried out within the framework of two research projects: an inter-SIRIC project (Socrate, Brio, Curamus, Lyrican) “Prescription Genomic Medicine” and an INCa project “Making sense of cancer.”

^3^Molecular Tumor Board are multidisciplinary meetings in which clinicians, biologists, clinical research associates and bioinformaticians participate. Their objective is either to refer a patient for a sequencing or to analyze the molecular results of the sequencing, in order to prescribe a treatment to the patient.

^4^ https://pfmg2025.aviesan.fr/professionnels/preindications-et-mise-en-place/cancers-avances-en-echec-therapeutique/

^5^The RMH (Royal Marsden Hospital) score is a biological test used to assess the patient's health status.

^6^Human Phenotype Ontology is a standardized vocabulary used for phenotype-driven differential diagnostics. It contains over 13,000 terms of phenotypic abnormalities that have been seen in human disease.

^7^Excerpt from the observation notebook of a workday of a prescription assistant in a health care facility in Parisian region, conducted on March 22, 2022.
